# Predictors of long-term clinical response in patients with non-radiographic axial spondyloarthritis receiving certolizumab pegol

**DOI:** 10.1186/s13075-021-02650-4

**Published:** 2021-10-29

**Authors:** Walter P. Maksymowych, Thomas Kumke, Simone E. Auteri, Bengt Hoepken, Lars Bauer, Martin Rudwaleit

**Affiliations:** 1 University of Alberta, Edmonton, Canada; 2 UCB Pharma, Monheim am Rhein, Germany; 3 UCB Pharma, Milan, Italy; 4 University of Bielefeld, Klinikum Bielefeld, Bielefeld, Germany

**Keywords:** TNF inhibitor, Axial spondyloarthritis, Predictors, Certolizumab pegol

## Abstract

**Background:**

Identification of predictive clinical factors of long-term treatment response may contribute to improved management of non-radiographic axSpA (nr-axSpA) patients. This analysis aims to identify whether any baseline characteristics or Week 12 clinical outcomes in nr-axSpA patients with elevated C-reactive protein (CRP) and/or sacroiliitis on magnetic resonance imaging (MRI) enrolled in the C-axSpAnd study are predictive of achieving clinical response after 1 year of certolizumab pegol (CZP).

**Methods:**

C-axSpAnd (NCT02552212) was a phase 3, multicentre study, including a 52-Week double-blind, placebo-controlled period. Enrolled patients were randomised to CZP 200 mg Q2W or placebo. Predictors of Week 12 (CZP group only) and Week 52 clinical response were identified using a multivariate stepwise logistic regression analysis. Response variables included Ankylosing Spondylitis Disease Activity Score major improvement (ASDAS-MI), Assessment of SpondyloArthritis International Society 40% response (ASAS40), Bath Ankylosing Spondylitis Disease Activity Index 50% response (BASDAI50) and ASDAS inactive disease (ASDAS-ID). Predictive factors assessed included demographic and baseline characteristics and clinical outcomes at Week 12. A *p*-value <0.05 was required for forward selection into the model and *p* ≥0.1 for backward elimination. Missing data or values collected after switching to open-label treatment were accounted for using non-responder imputation. Sensitivity analyses accounted for patients with changes in non-biologic background medication.

**Results:**

Of 317 enrolled patients, 159 and 158 were randomised to CZP and placebo, respectively. Younger age and male sex were identified as predictors of Week 12 response across all assessed efficacy outcomes in CZP-treated patients. Consistent predictors of Week 52 response, measured by ASDAS-MI, ASAS40 and BASDAI50, included human leukocyte antigen (HLA)-B27 positivity and sacroiliitis on MRI at baseline. MRI positivity was also predictive of achieving ASDAS-ID at Week 52. Sensitivity analyses were generally consistent with the primary analysis. In placebo-treated patients, no meaningful predictors of Week 52 response were identified.

**Conclusions:**

In this 52-Week, placebo-controlled study in nr-axSpA patients with elevated CRP and/or active sacroiliitis on MRI at baseline, MRI sacroiliitis and HLA-B27 positivity, but not elevated CRP or responses at Week 12, were predictive of long-term clinical response to CZP. Findings may support rheumatologists to identify patients suitable for TNFi treatment.

**Trial registration:**

ClinicalTrials.gov, NCT02552212. Registered on 15 September 2015

**Supplementary Information:**

The online version contains supplementary material available at 10.1186/s13075-021-02650-4.

## Background

Diagnosis can often be a challenge in non-radiographic axial spondyloarthritis (nr-axSpA); the presence of sacroiliitis on magnetic resonance imaging (MRI), human leukocyte antigen (HLA)-B27 and acute phase reactants such as serum C-reactive protein (CRP) remain the most reliable diagnostic tests but with limited specificity and/or sensitivity [1–3]. Patients with nr-axSpA are less likely to be treated with a biologic than patients with radiographic (r)-axSpA (also known as ankylosing spondylitis), despite a similar burden of disease on quality of life [4]. This may be due to the barriers associated with nr-axSpA diagnosis. Up to 25% of patients with nr-axSpA in the USA may face diagnostic delays of ≥11 years, during which time radiographic progression may occur [5, 6].

Personalised therapy using molecular and genetic profiling represents the optimal goal in the management of axSpA. The ASAS-EULAR task force highlights the value of predicting treatment response in axSpA and proposes that patients with high disease activity and objective signs of inflammation, defined as sacroiliac joint (SIJ) inflammation on MRI (MRI+) or elevated CRP level (CRP+), are suitable candidates for TNFi therapy [7, 8]. This is, in part, based on findings from randomised controlled trials in nr-axSpA, which suggested a better response to tumour necrosis factor inhibitor (TNFi) treatment in subgroups of patients with elevated CRP and/or MRI at baseline. However, it should be noted that these recommendations rely on non-significant outcomes, with primary endpoints reported for patients randomised to placebo or TNFi for a maximum of 12 or 16 Weeks, respectively [9–11].

Younger age, male sex, elevated CRP, HLA-B27 positivity and lower Bath Ankylosing Spondylitis Functional Index (BASFI) scores at baseline have been identified as predictors of good clinical response to TNFi across clinical trials and observational historical cohort studies in r-axSpA after 3–6 months of treatment [12–18]. However, there are fewer predictive analyses in nr-axSpA, with available data limited to short-term treatment responses only [9–11, 19]. The identification of predictive clinical factors of long-term response in nr-axSpA may help to differentiate appropriate patients suitable for TNFi treatment, while providing confidence in the treatment choice from both a physician and patient perspective, particularly in the early disease phase.

C-axSpAnd (NCT02552212) was a 52-Week placebo-controlled study to investigate the efficacy of a TNFi in a nr-axSpA population and resulted in the FDA approval of certolizumab pegol (CZP) as the first and currently only TNFi indicated for the treatment of nr-axSpA in the USA [20, 21]. All enrolled patients had elevated CRP and/or MRI and high disease activity despite prior non-steroidal anti-inflammatory drug (NSAID) use. C-axSpAnd was therefore designed to investigate CZP in patients with nr-axSpA, comparing the clinical benefit of a TNFi treatment with non-biologic background medication (NBBM) [21]. This post hoc analysis from C-axSpAnd aims to identify whether any baseline characteristics or Week 12 clinical outcomes in patients with nr-axSpA are predictive of achieving a clinical response after 1 year of CZP or placebo treatment.

## Patients and methods

### Study design

C-axSpAnd (AS0006, ClinicalTrials.gov number NCT02552212) was a 3-year, phase 3 study, including a 52-Week, randomised, placebo-controlled, double-blind period, followed by a 2-year open-label follow-up extension [21]. The aim of the study was to investigate the clinical efficacy and safety of CZP in patients with active nr-axSpA and elevated CRP and/or MRI compared with placebo and on top of NBBM, such as corticosteroids. The primary endpoint of the study was the achievement of Ankylosing Spondylitis Disease Activity Score major improvement (ASDAS-MI) at Week 52, defined as a reduction of ≥2.0 points in ASDAS relative to baseline, or when the lowest possible score was achieved (0.6) [21, 22]. This post hoc analysis aims to identify predictive factors of long-term clinical response to CZP or placebo in these patients.

Eligible patients were ≥18 years of age, had documented adult-onset nr-axSpA, fulfilling Assessment of SpondyloArthritis International Society (ASAS) classification criteria [23], with active disease, symptom duration of ≥12 months and previous inadequate response or contraindication to ≥2 NSAIDs. Patients were also required to have elevated CRP and/or active sacroiliitis on MRI (based on the ASAS/Outcome Measures in Rheumatology [OMERACT] definition of a positive MRI [MRI+]) [24] at screening and/or elevated CRP levels (CRP+) at baseline, which were above the upper limit of normal (ULN, ≥10.0 mg/L). Patients with radiographic sacroiliitis meeting the modified New York (mNY) classification criteria [25], as well as exposure to >1 TNFi prior to baseline or primary failure to any TNFi therapy, were excluded. All MRI and SIJ X-rays were centrally assessed by two readers and an adjudicator (if necessary). Assessment of CRP was performed in a central laboratory.

Eligible patients were randomised 1:1 to either CZP 200 mg Q2W (400 mg loading dose at Weeks 0, 2 and 4) or placebo; all patients received randomised treatment (CZP or placebo) on top of any NBBM. NBBM could be adjusted at the discretion of the investigator, although preferably not before Week 12, or within 4 Weeks prior to Weeks 24 and 52. Permitted concomitant medications are listed in the Supplementary Material. Switching to open-label CZP or alternative treatment was permitted at any point during the study. However, patients were encouraged to remain on randomised treatment until at least Week 12.

Full C-axSpAnd study design, as well as primary and secondary results, are reported elsewhere [21].

### Predictor analysis

Univariate analysis and subsequent multivariate logistic regression with stepwise variable selection was used to identify predictors of Week 12 and Week 52 response in the CZP treatment groups for the following efficacy outcomes: ASDAS-MI, ASAS 40% response (ASAS40), Bath Ankylosing Spondylitis Disease Activity Index 50% response (BASDAI50) and ASDAS inactive disease (ASDAS-ID [ASDAS <1.3]). All efficacy outcomes at Week 52 were also assessed for patients randomised to placebo.

Demographic and baseline characteristics, as well as Week 12 clinical outcomes (for the Week 52 analysis only), were included in the predictive model as either continuous or categorical variables. CRP level, MRI and HLA-B27 status were assessed as variables of special interest (see Supplementary Material for a full list of variables assessed). Smoking status was not included in the model as a potential predictor of response due to the small number of smokers enrolled in the trial (CZP: *n* = 27; placebo: *n* = 18). Variables were initially assessed in a univariate analysis, with significant results subsequently included in the multivariate selection model. A *p*-value <0.05 was required for forward selection into the model and *p* ≥0.1 for backward elimination from the model. The final logistic model included collinearity diagnostics. All patients who were randomised into the study (Randomised Set) were included in the analysis. Non-responder imputation was used to account for missing data or values collected after switching to open-label treatment.

A sensitivity analysis was conducted to identify predictors of response for all four response variables, excluding patients who had changes in NBBM during the 52-Week placebo-controlled phase. An additional analysis was also performed to investigate interaction effects in the Week 12 and Week 52 models. The following interaction effect variables were considered: MRI*HLA-B27, sex*MRI, age*MRI, CRP*MRI, sex*CRP and age*CRP.

Predictive factors are reported as odds ratios (OR) with 95% confidence intervals (CI). Statistical analyses were performed using SAS version 9.4 or above.

## Results

### Patients

A total of 317 patients with nr-axSpA and elevated CRP and/or active sacroiliitis on MRI (MRI positive or MRI negative) were enrolled in C-axSpAnd and were randomised to either CZP 200 mg Q2W (*n* = 159) plus NBBM or placebo (*n* = 158) plus NBBM. Of the 282 patients who completed the Week 52 visit, 180 remained on randomised double-blind treatment (CZP: *n* = 125; placebo: *n* = 55). Baseline demographics and characteristics were representative of a nr-axSpA patient population and were well-balanced between treatment groups (Table 1).

**Table 1:** Baseline demographics and patient characteristics

	Placebo + NBBM (***n*** = 158)	CZP 200 mg Q2W + NBBM (***n*** = 159)
Age (years), mean (*SD*)	37.4 (10.8)	37.3 (10.5)
Female, *n* (%)	82 (52)	81 (51)
HLA-B27 positive, *n* (%)	132 (84)	128 (81)
Caucasian, *n* (%)	148 (94)	152 (96)
Symptom duration (years), mean (*SD*)	8.0 (7.5)	7.8 (7.7)
Time since first diagnosis (years), mean (*SD*)	4.0 (5.4)	3.6 (4.8)
ASDAS, mean (*SD*)	3.8 (0.9)	3.8 (0.8)
BASDAI score, mean (*SD*)	6.8 (1.3)	6.9 (1.4)
BASFI score, mean (*SD*)	5.4 (2.2)	5.4 (2.1)
BASMI score, mean (*SD*)	2.8 (1.4)	3.0 (1.3)
CRP (mg/L), mean (*SD*)	15.8 (17.7)	15.8 (17.8)
Elevated CRP at baseline (>ULN), *n* (%)	83 (53)	89 (56)
MRI/CRP classification, *n* (%)		
MRI+/CRP+	42 (27)	45 (28)
MRI+/CRP−	76 (48)	74 (47)
MRI−/CRP+	39 (25)	38 (24)
Sacroiliac joint MRI SPARCC score, mean (*SD*)	8.5 (12.3)	7.8 (10.8)
Sacroiliac joint MRI SPARCC score ≥2, *n* (%)	129 (82)	136 (86)
Nocturnal spinal pain score, mean (*SD*)	6.6 (2.1)	6.6 (2.3)
Past treatment,^a^ *n* (%)		
NSAIDs	144 (91)	143 (90)
DMARDs^b^	46 (29)	44 (28)
Tumour necrosis factor inhibitors	11 (7)	7 (4)
Concomitant treatment, *n* (%)		
NSAIDs	138 (87)	138 (87)
DMARDs	48 (30)	55 (35)
Systemic corticosteroids	26 (17)	27 (17)

Randomised set (*N* = 317). All patient-reported outcomes were assessed using a numerical rating scale (0–10), with higher numbers indicating poorer outcomes. ^a^Medications with an end date before the baseline visit; ^b^includes biologic DMARDs and conventional synthetic DMARDs. *ASDAS* Ankylosing Spondylitis Disease Activity Score, *BASDAI* Bath Ankylosing Spondylitis Disease Activity Index, *BASFI* Bath Ankylosing Spondylitis Functional Index, *BASMI* Bath Ankylosing Spondylitis Metrology Index, *CRP* C-reactive protein, *CZP* certolizumab pegol, *DMARD* disease-modifying antirheumatic drug, *HLA-B27* human leukocyte antigen B27, *MRI* magnetic resonance imaging, *NBBM* non-biologic background medication, *NSAID* non-steroidal anti-inflammatory drug, *Q2W* every 2 Weeks, *SD* standard deviation, *SPARCC* Spondyloarthritis Research Consortium of Canada, *ULN* upper limit of normal (9.99 mg/L)

### Predictors of Week 12 response

**Fig. 1:**
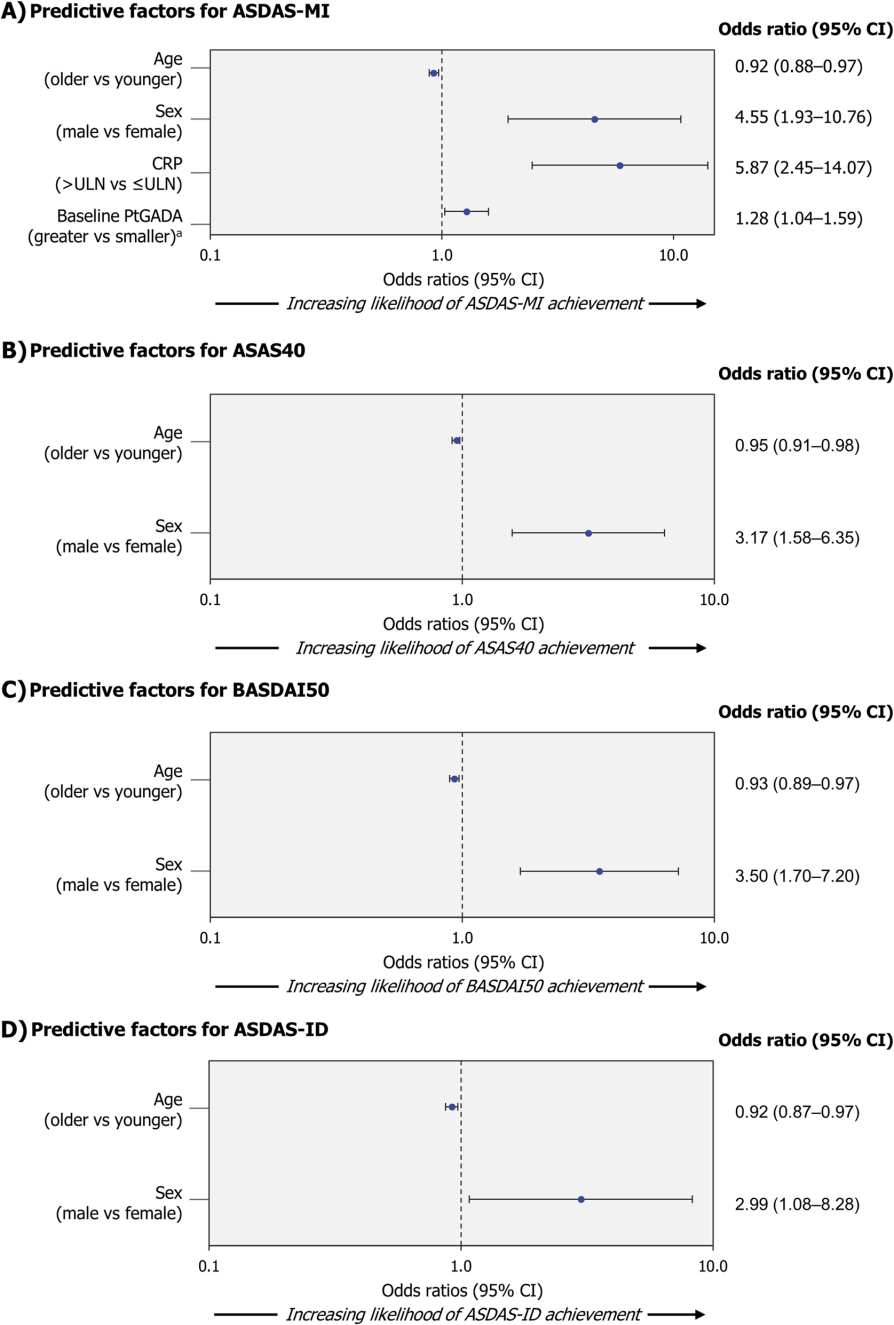
Predictive factors of Week 12 response in CZP-treated patients. Randomised set (NRI). Patients received CZP 200 mg Q2W (400 mg loading dose at Weeks 0, 2 and 4) plus non-biologic background medication. ^a^Included in the predictive model as continuous variables; for these factors, an odds ratio >1 indicates a higher probability of larger values being predictive of a response. ASAS40 Assessment of SpondyloArthritis International Society 40%, ASDAS Ankylosing Spondylitis Disease Activity Score, ASDAS-ID ASDAS inactive disease (ASDAS <1.3), ASDAS-MI ASDAS major improvement (reduction in ASDAS ≥2.0), BASDAI50 Bath Ankylosing Spondylitis Disease Activity Index 50%, CI confidence interval, CRP C-reactive protein, CZP certolizumab pegol, NRI non-responder imputation, PtGADA Patient’s Global Assessment of Disease Activity, Q2W every 2 Weeks, ULN upper limit of normal (9.99 mg/L), vs versus

The percentages of patients achieving an ASDAS-MI, ASAS40, BASDAI50 or ASDAS-ID response at Week 12 are reported in Table 2. Younger age and male sex were associated with all analysed response variables (ASDAS-MI, ASAS40, BASDAI50 and ASDAS-ID) at Week 12 for CZP-treated patients (Fig. 1). Achievement of ASDAS-MI at Week 12 was also associated with elevated CRP levels and higher Patient Global Assessment of Disease Activity (PtGADA) at baseline (Fig. 1A). Univariate analyses are reported in Supplementary Figure S1. Variance inflation factors ranged between 1.02 and 1.06 and tolerance ranged between 0.94 and 0.98, indicating that potential bias due to multicollinearity is negligible.

**Table 2:** Percentage of patients achieving a response at Week 12 and Week 52 (NRI)

*n (%)*	Week 12		Week 52	
	*Placebo* + NBBM *(n = 158)*	*CZP 200 mg Q2W* + NBBM *(n = 159)*	*Placebo* + NBBM *(n = 158)*	*CZP 200 mg Q2W* + NBBM *(n = 159)*
**ASDAS-MI**	10 (6.3)	56 (35.2)	11 (7.0)	75 (47.2)
**ASAS40**	18 (11.4)	76 (47.8)	25 (15.8)	90 (56.6)
**BASDAI50**	23 (14.6)	68 (42.8)	19 (12.0)	88 (55.3)
**ASDAS-ID**	2 (1.3)	26 (16.4)	6 (3.8)	44 (27.7)

*ASAS40* Assessment of SpondyloArthritis International Society 40%, *ASDAS* Ankylosing Spondylitis Disease Activity Score, *ASDAS-ID* ASDAS inactive disease (ASDAS <1.3), *ASDAS-MI* ASDAS major improvement (reduction in ASDAS ≥2.0), *BASDAI50* Bath Ankylosing Spondylitis Disease Activity Index 50%, *NRI* non-responder imputation, *Q2W* every 2 Weeks

### Predictors of Week 52 response

**Fig. 2:**
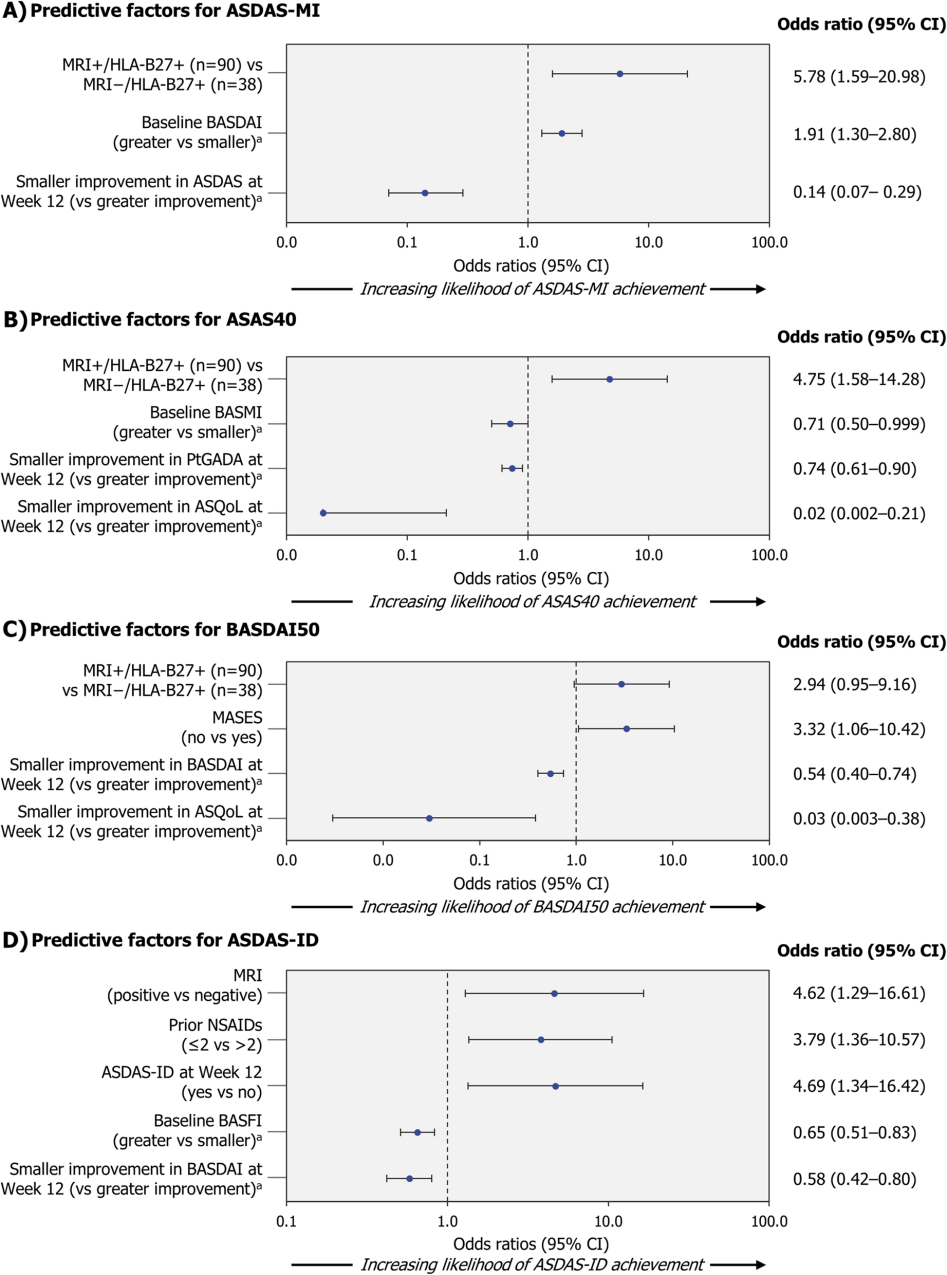
Predictive factors of Week 52 response in CZP-treated patients. Randomised set (NRI). Patients received CZP 200 mg Q2W (400 mg loading dose at Weeks 0, 2 and 4) plus non-biologic background medication. ^a^Included in the predictive model as continuous variables; for these factors, an odds ratio >1 indicates a higher probability of larger values being predictive of a response. For Week 12 change from baseline measures, a lower (negative) value is indicative of improvement, while larger (positive) values indicate worsening. ASAS40 Assessment of SpondyloArthritis International Society 40%, ASDAS Ankylosing Spondylitis Disease Activity Score, ASDAS-ID ASDAS inactive disease (ASDAS<1.3), ASDAS-MI ASDAS major improvement (reduction in ASDAS≥2.0), ASQoL ankylosing spondylitis quality of life, BASDAI50 Bath Ankylosing Spondylitis Disease Activity Index 50%, BASFI Bath Ankylosing Spondyloarthritis Functional Index, BASMI Bath Ankylosing Spondylitis Metrology Index, CI confidence interval, CZP certolizumab pegol, HLA-B27 human leukocyte antigen-B27, MRI+/− presence/absence of sacroiliitis on magnetic resonance imaging, MASES Maastricht Ankylosing Spondylitis Enthesitis Score (range 0−13), NRI non-responder imputation, NSAID non-steroidal anti-inflammatory drug, PtGADA Patient Global Assessment of Disease Activity, Q2W every 2 Weeks, vs versus

The percentages of patients achieving an ASDAS-MI, ASAS40, BASDAI50 or ASDAS-ID response at Week 52 are reported in Table 2. Predictive factors identified for Week 52 ASDAS-MI in CZP-treated patients included being positive for both presence of sacroiliitis on MRI and HLA-B27, having a higher BASDAI at baseline and having a greater Week 12 improvement in ASDAS (Fig. 2A). For ASAS40 response, MRI+/HLA-B27+ was also identified as a predictor of Week 52 response, along with a lower BASMI and greater Week 12 improvements in PtGADA and ankylosing spondylitis quality of life (ASQoL, Fig. 2B). Positive MRI/HLA-B27 status, greater Week 12 improvements in BASDAI and ASQoL, and a Maastricht Ankylosing Spondylitis Enthesitis Score (MASES) of 0 at baseline were associated with Week 52 BASDAI50 (Fig. 2C). Achievement of ASDAS-ID at Week 52 was associated with MRI positivity, prior exposure to ≤2 NSAIDs, lower BASFI at baseline and greater BASDAI Week 12 improvement. Reaching ASDAS-ID at Week 12 also increased the likelihood of having ASDAS-ID at Week 52 (Fig. 2D). Univariate analyses for Week 52 responses in CZP-treated patients are reported in Supplementary Figure S2.

No meaningful predictors of Week 52 response were identified in placebo-randomised patients; there were no outputs for the multivariate analysis as data were non-significant (data not shown). A summary of Week 12 and Week 52 predictive factors of response in CZP-treated patients is shown in Table 3.

**Table 3:** Summary of Week 12 and Week 52 predictive factors of response in CZP-treated patients

Response	Week 12	Week 52
**ASDAS-MI**	- Younger age - Male sex - Elevated CRP - Higher PtGADA at baseline	- MRI+/HLA-B27+ - Higher BASDAI at baseline - Greater Week 12 ASDAS improvement
**ASAS40**	- Younger age - Male sex	- MRI+/HLA-B27+ - Lower BASMI at baseline - Greater Week 12 PtGADA and ASQoL improvements
**BASDAI50**	- Younger age - Male sex	- MRI+/HLA-B27+ - MASES = 0 - Greater Week 12 BASDAI and ASQoL improvements
**ASDAS-ID**	- Younger age - Male sex	- Positive MRI - ≤2 prior NSAIDs - ASDAS-ID at Week 12 - Lower BASFI at baseline - Greater Week 12 BASDAI improvement

*ASAS40* Assessment of SpondyloArthritis International Society 40%, *ASDAS* Ankylosing Spondylitis Disease Activity Score, *ASDAS-ID* ASDAS inactive disease (ASDAS <1.3), *ASDAS-MI* ASDAS major improvement (reduction in ASDAS ≥2.0), *ASQoL* Ankylosing Spondylitis Quality of Life, *BASDAI50* Bath Ankylosing Spondylitis Disease Activity Index 50%, *BASMI* Bath Ankylosing Spondylitis Metrology Index, *HLA-B27* human leukocyte antigen-B27, *MRI* magnetic resonance imaging, *MASES* Maastricht Ankylosing Spondylitis Enthesitis Score (range 0−13), *NSAID* non-steroidal anti-inflammatory drug, *PtGADA* Patient Global Assessment of Disease Activity

Variance inflation factors ranged between 1.00 and 1.65 and tolerance ranged from 1.00 to 0.60, indicating a slight presence of multicollinearity.

### Sensitivity analysis

A sensitivity analysis accounting for changes in background medication identified the same predictors for ASDAS-MI and ASAS40, with the exception of change from baseline in PtGADA as a predictor of ASAS40. Sensitivity analysis also identified achievement of Week 12 ASAS40 as a predictor of Week 52 ASAS40 (Supplementary Figure S3). Sensitivity analysis for Week 52 BASDAI50 was generally consistent with the primary analysis, excluding low MASES (MASES = 0) at baseline. ASDAS-ID at Week 12 and lower BASFI at baseline were identified as predictors of Week 52 ASDAS-ID in the sensitivity analysis, in addition to positive MRI/HLA-B27 status and BASDAI50 at Week 12. MRI positivity (as a dichotomous measure) and prior NSAID exposure were not significantly associated with Week 52 ASDAS-ID in CZP-treated patients who did not experience changes in background medication.

### Interaction effects analysis

The results from the interaction effects analysis were mostly non-significant. Significant interaction effects were identified between MRI and HLA-B27 status for Week 52 ASDAS-MI, ASAS40, BASDAI50 and ASDAS-ID responses (Table 4). A significant association between sex and MRI was also identified for Week 52 ASDAS-MI and ASDAS-ID responses (Table 4).

**Table 4:** Interaction effects analysis for the Week 12 and Week 52 predictor models

	Week 12				Week 52			
Interaction effect	ASDAS-MI	ASAS40	BASDAI50	ASDAS-ID	ASDAS-MI	ASAS40	BASDAI50	ASDAS-ID
MRI*HLA-B27	0.5599	0.8460	0.1517	0.6183	0.0035*	0.0014*	0.0049*	0.0247*
Sex*MRI	0.6680	0.3600	0.6663	0.9666	0.0005*	0.9107	0.1873	0.0030*
Age*MRI	0.8676	0.5666	0.0916	0.2039	0.9140	0.8291	0.4061	0.7247
CRP*MRI	0.8584	0.4112	0.7003	0.0681	0.0626	0.7511	0.7955	0.4636
Sex*CRP	0.7179	0.3568	0.5063	0.8552	0.8904	0.7033	0.6215	0.2282
Age*CRP	0.9958	0.4325	0.0985	0.5441	0.0526	0.3986	0.9907	0.3007

*p*-values from the final regression step are reported. *Significant interaction terms included in the final model. *ASAS40* Assessment of SpondyloArthritis International Society 40%, *ASDAS* Ankylosing Spondylitis Disease Activity Score, *ASDAS-ID* ASDAS inactive disease (ASDAS <1.3), *ASDAS-MI* ASDAS major improvement (reduction in ASDAS ≥2.0), *BASDAI50* Bath Ankylosing Spondylitis Disease Activity Index 50%, *HLA-B27* human leukocyte antigen-B27, *MRI* magnetic resonance imaging

## Discussion

In this report from a double-blinded, 52-Week placebo-controlled study, we investigated predictors of clinical response in a nr-axSpA population with objective signs of inflammation by elevated CRP and/or positive MRI. Specifically, this analysis aimed to establish whether any demographic and baseline characteristics or clinical outcomes at Week 12 are predictive of achieving long-term clinical response to CZP in patients with nr-axSpA and elevated CRP and/or active sacroiliitis on MRI, thereby supporting rheumatologists to identify patients suitable for TNFi treatment.

In the Week 12 analysis, younger age and male sex were identified as consistent predictors of response across all clinical outcomes, including ASDAS-MI, ASAS40, BASDAI50 and ASDAS-ID. This is similar to another analysis, which also identified younger age and male sex as predictors of ASDAS-ID response and ASAS partial remission in patients with nr-axSpA who were treated with adalimumab over a 28-Week open-label lead-in period [26]. The finding that younger patients and males were associated with predictors of Week 12 but not Week 52 response in this analysis may indicate a delayed or weaker treatment response in females and older individuals; however, this remains unclear. Several studies have identified a significant difference in TNFi treatment response between male and female patients with r-axSpA, which may be attributed to possible differences in immunological, hormonal and genetic responses [27]. Additional data in nr-axSpA patients have also been reported supporting this observation. These findings may highlight the challenges of making an accurate diagnosis of nr-axSpA in females and the potential for disease misclassification in these patients [28].

Consistent predictors of Week 52 response in CZP-treated patients, as measured by ASDAS-MI, ASAS40 and BASDAI50, included HLA-B27 positivity and presence of sacroiliitis on MRI at baseline. MRI positivity was also predictive of achieving ASDAS-ID at Week 52. It is notable that these predictive factors were all objective features of disease and generally demonstrated stronger associations than the predictors of response identified for Week 12. Moreover, MRI scans were centrally read by trained readers, increasing confidence that true MRI sacroiliitis was identified. The use of SIJ MRI, in addition to clinical examination and assessment of CRP levels, is encouraged to aid the diagnosis of axSpA and in some countries is a mandatory requirement for the initiation of TNFi [7, 8]. Widespread inflammation on MRI, particularly in the spine, has been associated with a good clinical response to TNFi therapy in r-axSpA [12]. Although previous studies have evaluated the prognostic value of MRI in nr-axSpA [26], most findings are data extracted from correlation analyses [9, 29].

Overall, the findings of this analysis are comparable with analyses in r-axSpA, which have identified predictors of clinical response following up to 6 months of TNFi treatment [12–19]. However, in contrast to previous studies, elevated CRP (included as a categorical variable, ≤/> upper limit of normal) was not identified as a predictive factor for any evaluated efficacy outcome, except Week 12 ASDAS-MI. Compared with other predictive analyses, C-axSpAnd enrolled patients with elevated CRP and/or active sacroiliitis on MRI, which reflects current ASAS-EULAR recommendations for the initiation of TNFi therapy in axSpA [7, 8]. Patients included in this analysis therefore generally had greater baseline CRP levels than previously reported, which may explain why CRP was not identified as a predictor of long-term clinical response. It should also be noted that the demographics of patients with nr-axSpA are different from the r-axSpA populations previously studied, which may influence treatment response; for instance, patients with nr-axSpA are more likely to be female and tend to have shorter symptom duration [30].

An observational study in a cohort of patients with early axSpA has also demonstrated the limited predictive capacity of CRP, with sacroiliitis on MRI identified as the only predictor of TNFi response. However, compared with C-axSpAnd, patients were not selected for elevated MRI and/or CRP; eligible patients were required to have inflammatory back pain, with approximately 30% presenting with radiographic sacroiliitis meeting mNY classification criteria at baseline [31].

Compared with previous predictive analyses in axSpA, this analysis exhibits a number of strengths. Firstly, data are reported over a 52-Week treatment period, which is the longest time exposure with a biologic in a controlled setting, allowing the identification of long-term predictors of response. A greater number of potential predictors of response are also evaluated, including responses at Week 12, with variables such as age included in the predictive model as continuous variables, rather than using arbitrary cut-offs. MRI and HLA-B27 status are also included as both combined and individual variables in the model. Although it was not possible in this analysis due to the selection criteria, it would be interesting to evaluate clinical responses in a subgroup of patients who were MRI+/HLA-B27− as compared to MRI−/HLA-B27−. It should be noted that the patients with axSpA who were MRI−/HLA-B27− were excluded from the study, as dictated by ASAS classification criteria [23]. Stringent measures of axSpA disease activity were also used in this analysis, including ASDAS-ID and ASDAS-MI, which were assessed as clinical endpoints. However, it remains unclear whether some of the identified predictors, such as Week 12 ASQoL improvements, had a true impact on clinical response or were statistically related to the study endpoints.

## Conclusions

To our knowledge, this is the first report from an interventional 52-Week placebo-controlled study in nr-axSpA to identify disease-related features, particularly the presence of sacroiliac joint inflammation and HLA-B27 positivity, as being predictive of long-term clinical response to CZP treatment. These findings may support rheumatologists to identify patients suitable for TNFi treatment and inform future research within the biologic class.

## Supplementary Information

**Additional file 1: Supplementary Appendix**.

## Data Availability

Data from this manuscript may be requested by qualified researchers 6 months after product approval in the USA and/or Europe, or global development is discontinued, and 18 months after trial completion. Investigators may request access to anonymised IPD and redacted study documents which may include raw datasets, analysis-ready datasets, study protocol, blank case report form, annotated case report form, statistical analysis plan, dataset specifications and clinical study report. Prior to use of the data, proposals need to be approved by an independent review panel at www.Vivli.org, and a signed data sharing agreement will need to be executed. All documents are available in English only, for a pre-specified time, typically 12 months, on a password-protected portal.

## Abbreviations

ASAS40: Assessment of SpondyloArthritis International Society 40% response
ASQoL: Ankylosing spondylitis quality of life
ASDAS: Ankylosing Spondylitis Disease Activity Score
ASDAS-ID: ASDAS inactive disease
ASDAS-MI: ASDAS major improvement
axSpA: Axial spondyloarthritis
BASDAI50: Bath Ankylosing Spondyloarthritis Disease Activity Index 50% response
BASFI: Bath Ankylosing Spondyloarthritis Functional Index
CRP: C-reactive protein
CZP: Certolizumab pegol
FDA: Food and Drug Administration
HLA-B27: Human leukocyte antigen-B27
MASES: Maastricht Ankylosing Spondylitis Enthesitis Score
MRI: Magnetic resonance imaging
mNY: Modified New York
NBBM: Non-biologic background medication
nr-axSpA: Non-radiographic axSpA
NSAID: Non-steroidal anti-inflammatory drug
OMERACT: Outcome Measures in Rheumatology
PtGADA: Patient Global Assessment of Disease Activity
r-axSpA: Radiographic axSpA
SIJ: Sacroiliac joint
TNFi: Tumour necrosis factor inhibitor
ULN: Upper limit of normal
USA: United States of America
