# Supplementary material for: Predictors of long-term clinical response in patients with non-radiographic axial spondyloarthritis receiving certolizumab pegol
Source: Arthritis Res Ther. 2021 Oct 29;23:274. doi: 10.1186/s13075-021-02650-4 (PMC8556993; doi:10.1186/s13075-021-02650-4)
Supplement: Supplementary file 1 — Additional file 1: Supplementary Appendix. [file 13075_2021_2650_MOESM1_ESM.pdf]

SUPPLEMENTARY APPENDIX

Predictive factors evaluated in the prediction model:

Baseline demographics and characteristics

Age (years)
Sex (male/female)
BMI (<18.5, 18.5≥BMI<25, 25≥BMI<30, ≥30)
Symptom duration (<5 years, ≥5 years)
Time since diagnosis (<5 years, ≥5 years)
CRP (≤ULN, >ULN)
MRI (+,-)
HLA-B27 (+,-)
MRI/HLA-B27 combined (MRI+/HLA-B27+, MRI+/HLA-B27-, MRI-/HLA-B27+)
Swollen Joint Count>0 (yes, no)
MASES>0 (yes, no)
Prior DMARDs (yes, no)
Concomitant DMARDs at baseline (yes, no)
Prior use of NSAIDs (≤2, >2)
ASDAS
BASDAI
BASFI
BASMI
SI joint SPARCC Score
Patient global assessment of disease activity
Total spinal pain
Nocturnal pain
ASQoL

Week 12 responses

ASDAS-MI (yes, no)
ASDAS-ID (yes, no)
ASAS40 (yes, no)
BASDAI50 (yes, no)

Permitted concomitant medications:

Non-biologic background medication permitted during C-axSpAnd included:

- Non-steroidal anti-inflammatory drugs (including COX-2 inhibitors)
- Opioid and non-opioid analgesics (including, but not limited to paracetamol and opiates)
- Conventional synthetic disease-modifying antirheumatic drugs (including sulfasalazine, hydrochloroquine, methotrexate, leflunomide and azathioprine)
- Disease-modifying antirheumatic drugs (including cyclosporine, cyclophosphamide, mycophenolic acid and apremilast)
- Hyaluronic acid
- Oral and intravenous corticosteroids

Figure S1. Univariate analyses of Week 12 predictors of response in CZP-treated patients (n=159)

A) Predictive factors for ASDAS-MI

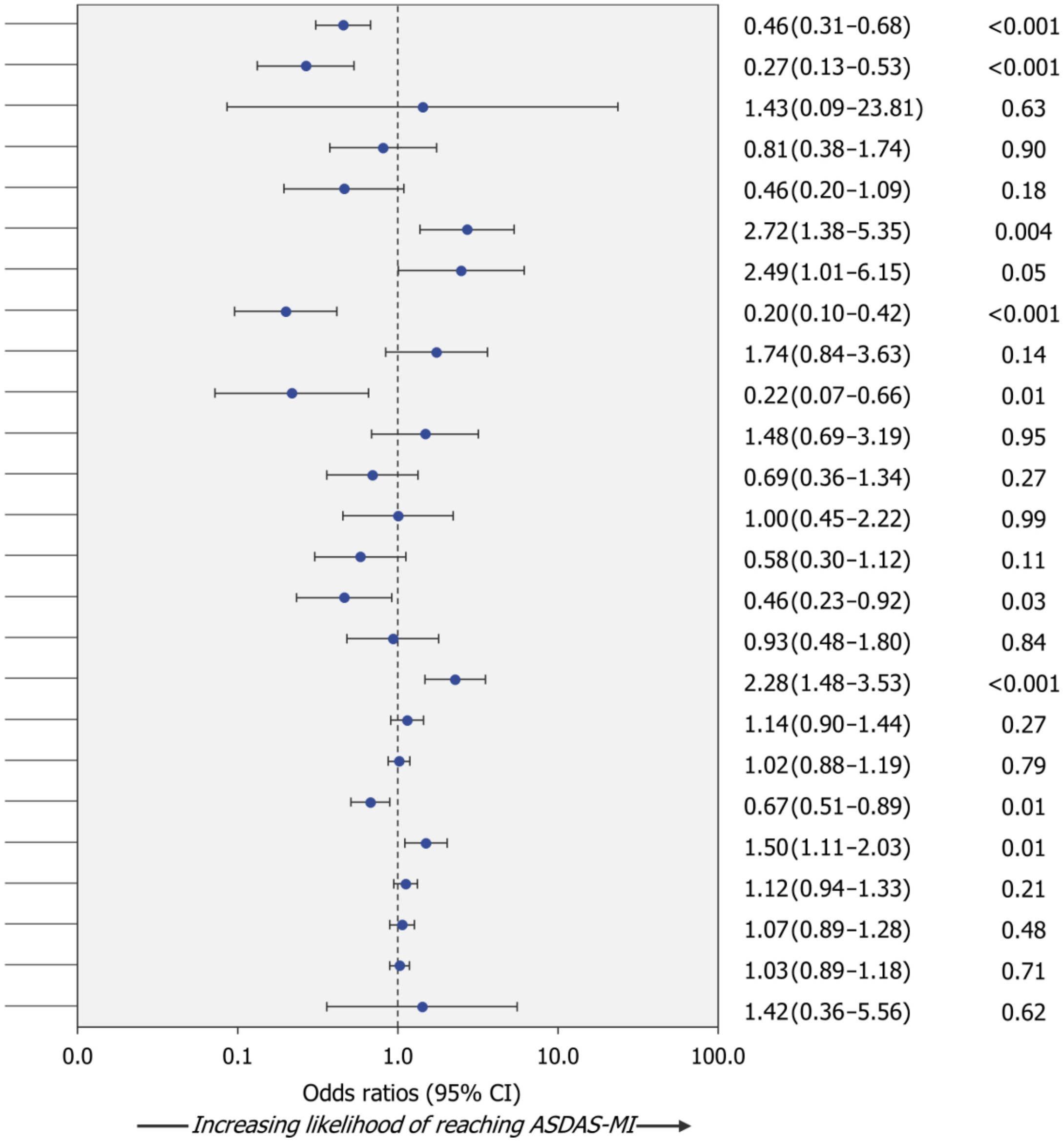

	Odds ratio (95% CI)	p-value
Age (older vs younger)	0.46(0.31–0.68)	<0.001
Gender (female vs male)	0.27(0.13–0.53)	<0.001
BMI (<18.5 vs 18.5<BMI<25)	1.43(0.09–23.81)	0.63
BMI (25≤BMI<30 vs 18.5<BMI<25)	0.81(0.38–1.74)	0.90
BMI (≥30 vs 18.5<BMI<25)	0.46(0.20–1.09)	0.18
Symptom duration (<5 years vs ≥5 years)	2.72(1.38–5.35)	0.004
Time since diagnosis (<5 years vs ≥5 years)	2.49(1.01–6.15)	0.05
CRP (≤ULN vs >ULN)	0.20(0.10–0.42)	<0.001
MRI (negative vs positive)	1.74(0.84–3.63)	0.14
HLA-B27 (negative vs positive)	0.22(0.07–0.66)	0.01
MRI-/HLA-B27+ vs MRI+/HLA-B27+	1.48(0.69–3.19)	0.95
Swollen joint count >0 (no vs yes)	0.69(0.36–1.34)	0.27
MASES>0 (no vs yes)	1.00(0.45–2.22)	0.99
Prior DMARDs (no vs yes)	0.58(0.30–1.12)	0.11
Concomitant DMARDs at baseline (no vs yes)	0.46(0.23–0.92)	0.03
Prior use of NSAIDs (≤2 vs >2)	0.93(0.48–1.80)	0.84
Baseline ASDAS (greater vs smaller)	2.28(1.48–3.53)	<0.001
Baseline BASDAI score (greater vs smaller)	1.14(0.90–1.44)	0.27
Baseline BASFI score (greater vs smaller)	1.02(0.88–1.19)	0.79
Baseline BASMI score (greater vs smaller)	0.67(0.51–0.89)	0.01
Baseline SI joint SPARCC score (greater vs smaller)	1.50(1.11–2.03)	0.01
Baseline PtGADA (greater vs smaller)	1.12(0.94–1.33)	0.21
Baseline total spinal pain score (greater vs smaller)	1.07(0.89–1.28)	0.48
Baseline nocturnal pain score (greater vs smaller)	1.03(0.89–1.18)	0.71
Baseline AsQoL (greater vs smaller)	1.42(0.36–5.56)	0.62

B) Predictive factors for ASAS40

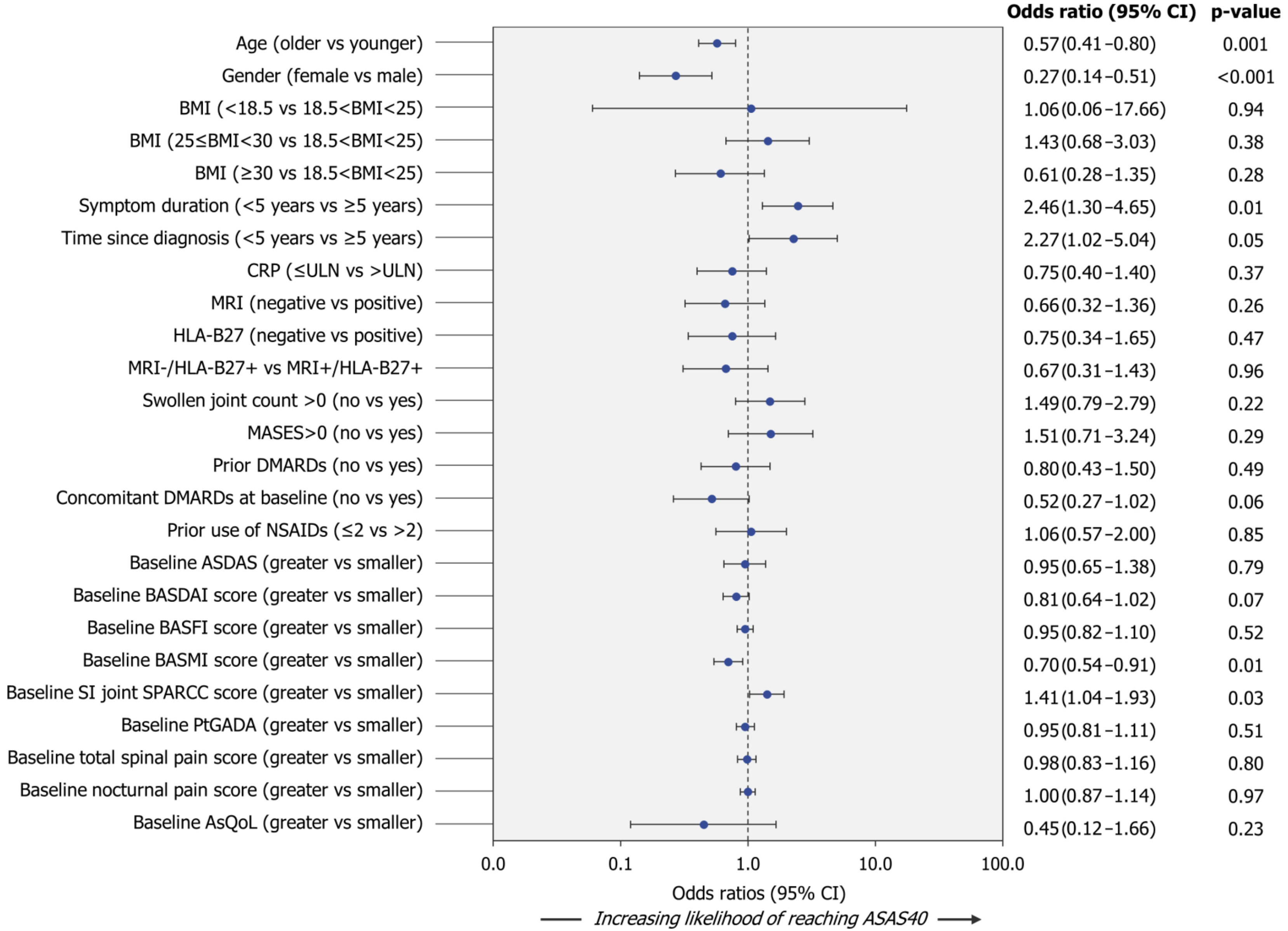

C) Predictive factors for BASDAI50

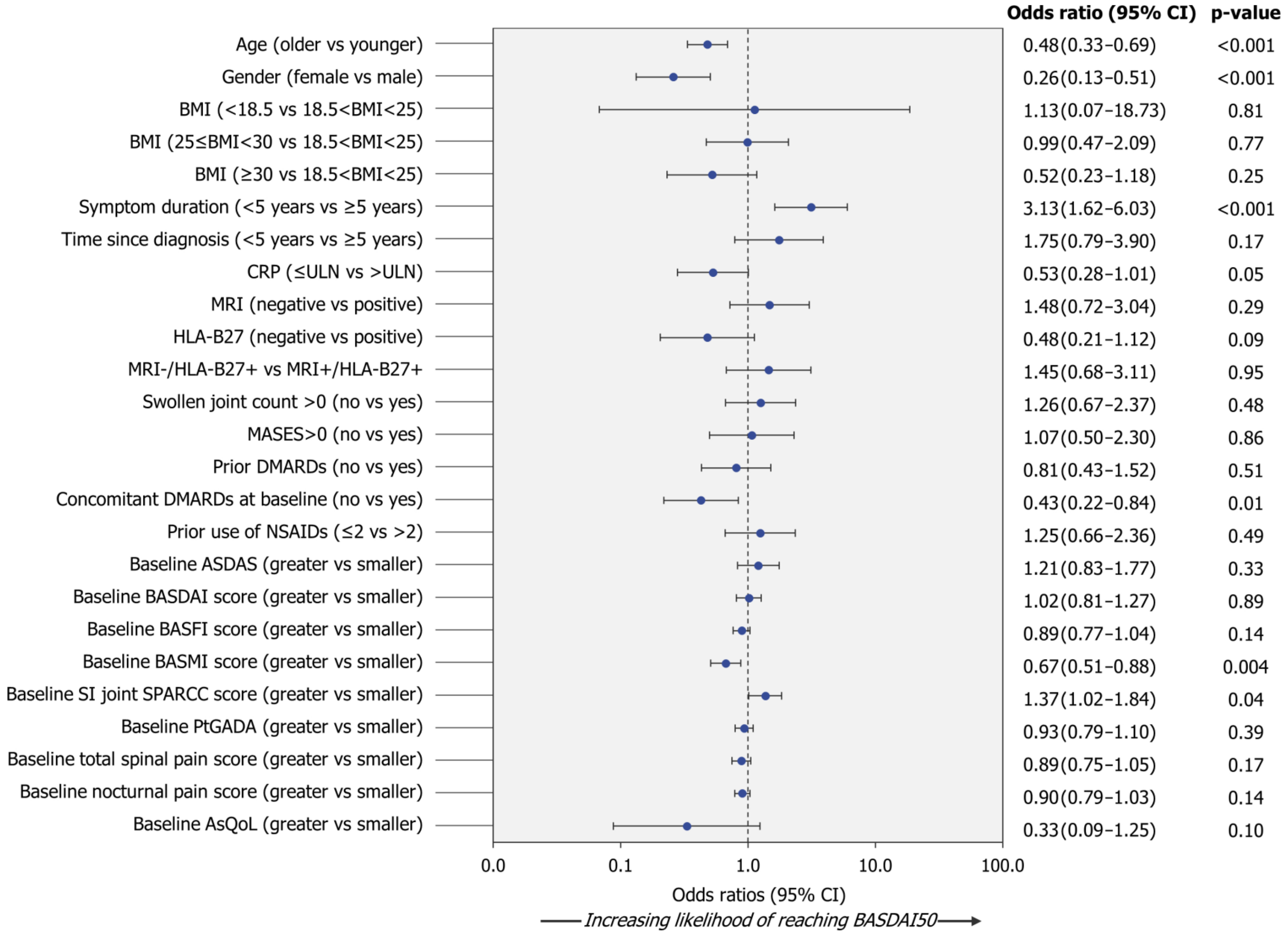

D) Predictive factors for ASDAS-ID

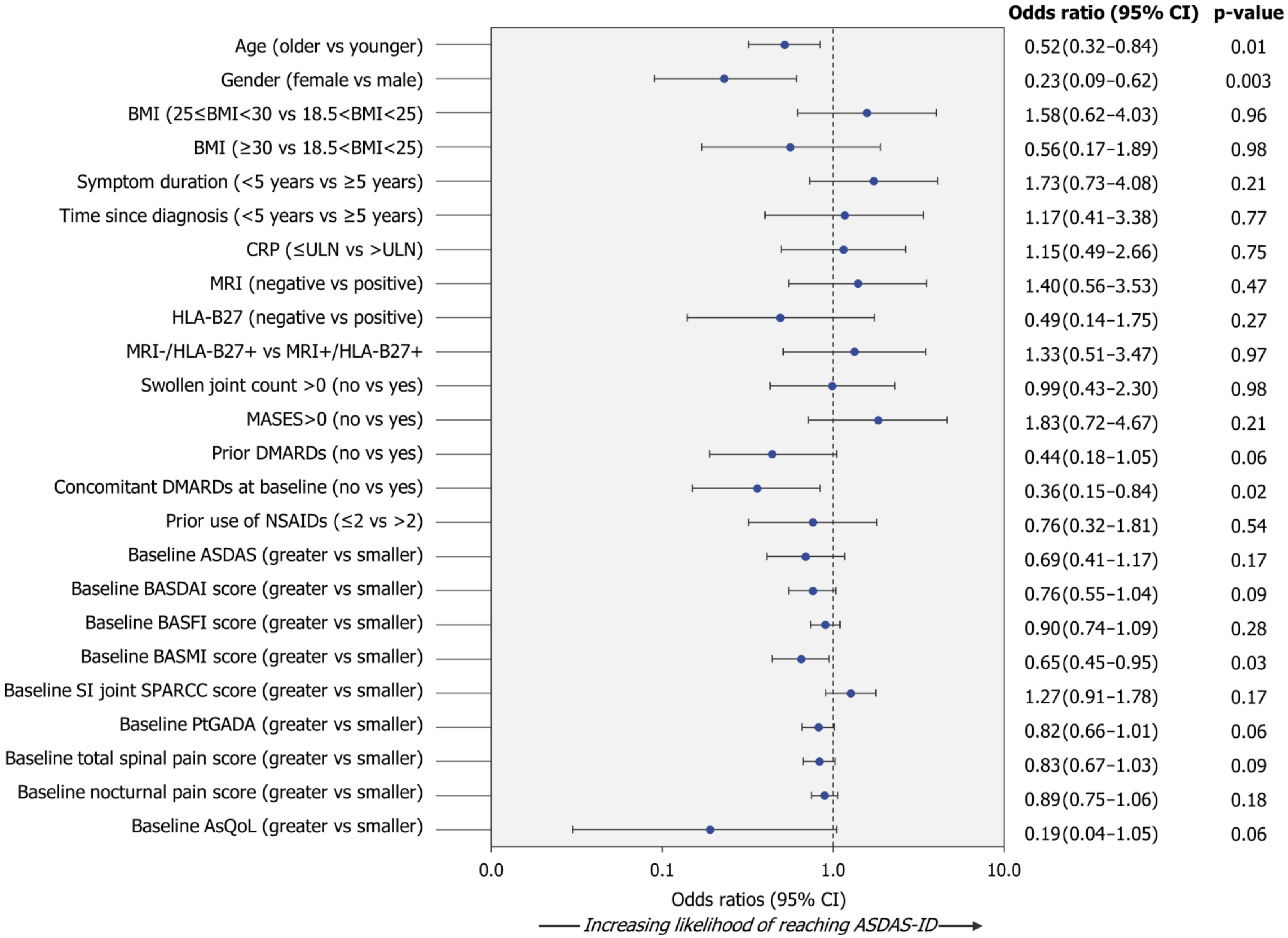

Full Analysis set (n=159). Patients received CZP 200 mg Q2W (400 mg loading dose at Weeks 0, 2 and 4) plus non-biologic background medication. Variables with 95% CI that were <0.0001 or >999.9 are excluded. 95% CI and p-values are considered non-confirmatory due to the exploratory nature of the analysis. ASAS40: Assessment of SpondyloArthritis International Society 40%; ASDAS: Ankylosing Spondylitis Disease Activity Score; ASDAS-ID: ASDAS inactive disease (ASDAS<1.3); ASDAS-MI: ASDAS major improvement (reduction in ASDAS≥2.0); ASQoL: ankylosing spondylitis quality of life; BASDAI50: Bath Ankylosing Spondylitis Disease Activity Index 50%; BASFI: Bath Ankylosing Spondylitis Functional Index; BASMI: Bath Ankylosing Spondylitis Metrology Index; BMI: body mass index; CI: confidence interval; CRP: C-reactive protein; CZP: certolizumab pegol; DMARD: disease-modifying antirheumatic drug; HLA-B27: human leukocyte antigen-B27; MRI+/-: presence/absence of sacroiliitis on magnetic resonance imaging; MASES: Maastricht Ankylosing Spondylitis Enthesitis Score (range 0−13); NRI: non-responder imputation; NSAID: non-steroidal anti-inflammatory drug; PtGADA: Patient Global Assessment of Disease Activity; Q2W: every 2 weeks; SPARCC: Spondyloarthritis Research Consortium of Canada; vs: versus.

Figure S2. Univariate analyses of Week 52 predictors of response in CZP-treated patients (n=159)

A) Predictive factors for ASDAS-MI

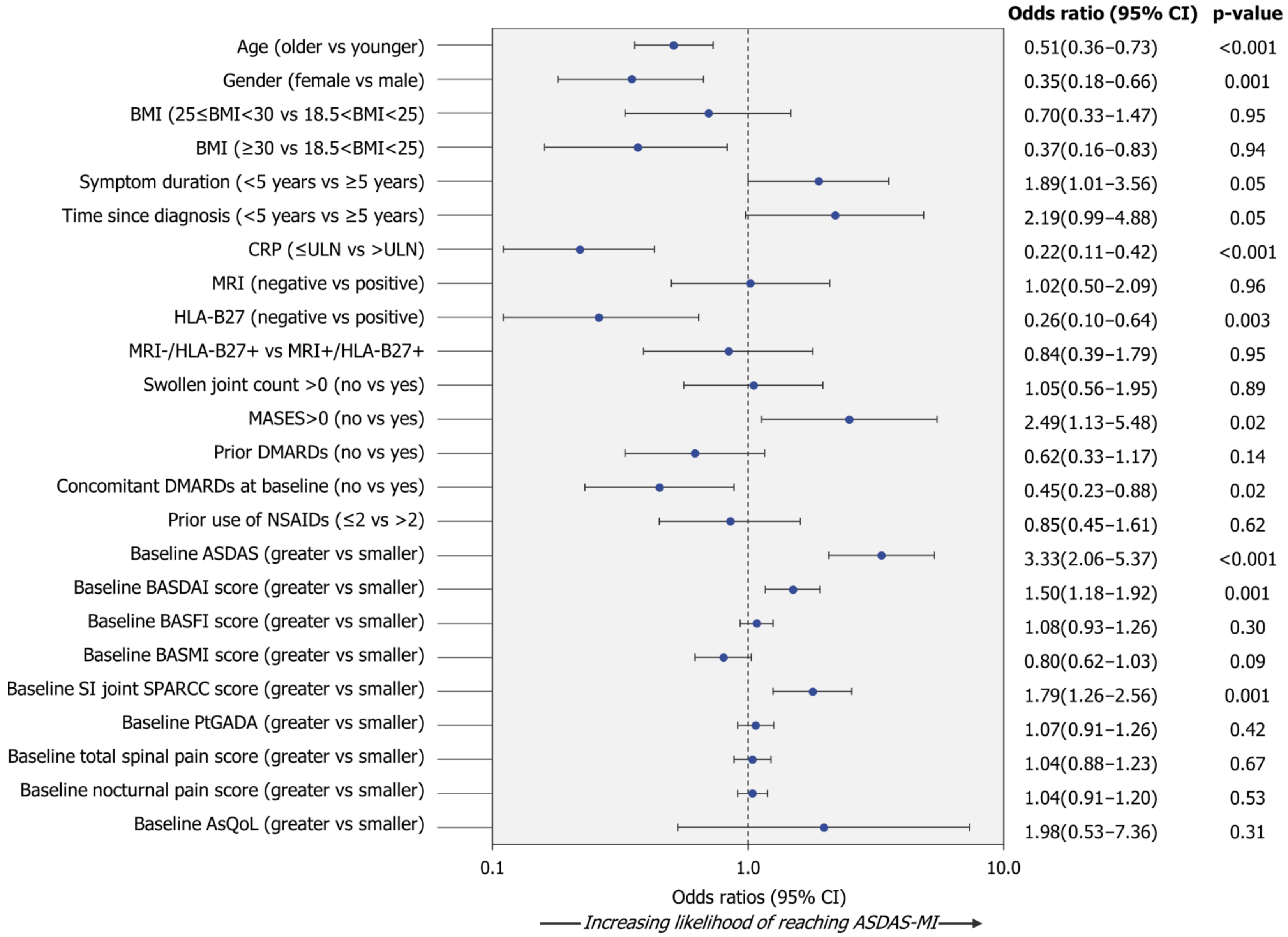

B) Predictive factors for ASAS40

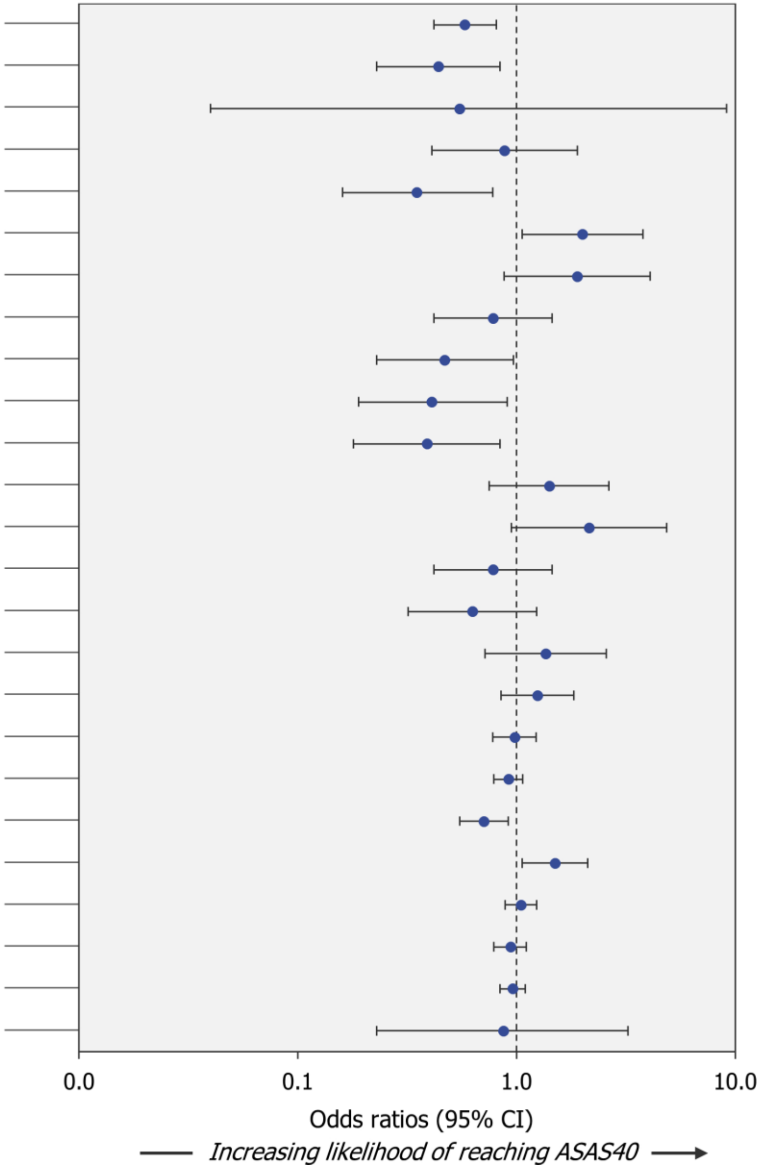

	Odds ratio (95% CI)	p-value
Age (older vs younger)	0.58(0.42–0.81)	0.001
Gender (female vs male)	0.44(0.23–0.84)	0.01
BMI (<18.5 vs 18.5<BMI<25)	0.55(0.03–9.12)	0.88
BMI (25≤BMI<30 vs 18.5<BMI<25)	0.88(0.41–1.90)	0.46
BMI (≥30 vs 18.5<BMI<25)	0.35(0.16–0.78)	0.17
Symptom duration (<5 years vs ≥5 years)	2.00(1.06–3.79)	0.03
Time since diagnosis (<5 years vs ≥5 years)	1.90(0.88–4.09)	0.10
CRP (≤ULN vs >ULN)	0.78(0.41–1.46)	0.43
MRI (negative vs positive)	0.47(0.23–0.96)	0.04
HLA-B27 (negative vs positive)	0.41(0.18–0.91)	0.03
MRI-/HLA-B27+ vs MRI+/HLA-B27+	0.39(0.18–0.84)	0.96
Swollen joint count >0 (no vs yes)	1.41(0.75–2.65)	0.29
MASES>0 (no vs yes)	2.15(0.95–4.86)	0.07
Prior DMARDs (no vs yes)	0.78(0.42–1.46)	0.44
Concomitant DMARDs at baseline (no vs yes)	0.63(0.32–1.23)	0.18
Prior use of NSAIDs (≤2 vs >2)	1.36(0.72–2.57)	0.35
Baseline ASDAS (greater vs smaller)	1.25(0.85–1.83)	0.26
Baseline BASDAI score (greater vs smaller)	0.98(0.79–1.23)	0.89
Baseline BASFI score (greater vs smaller)	0.92(0.79–1.07)	0.30
Baseline BASMI score (greater vs smaller)	0.71(0.55–0.92)	0.01
Baseline SI joint SPARCC score (greater vs smaller)	1.50(1.06–2.12)	0.02
Baseline PtGADA (greater vs smaller)	1.05(0.89–1.24)	0.55
Baseline total spinal pain score (greater vs smaller)	0.94(0.79–1.11)	0.44
Baseline nocturnal pain score (greater vs smaller)	0.96(0.84–1.10)	0.55
Baseline AsQoL (greater vs smaller)	0.87(0.23–3.23)	0.83

C) Predictive factors for BASDAI50

	Odds ratio (95% CI)	p-value
Age (older vs younger)	0.52(0.37–0.73)	<0.001
Gender (female vs male)	0.32(0.17–0.62)	<0.001
BMI (25≤BMI<30 vs 18.5<BMI<25)	0.86(0.40–1.84)	0.95
BMI (≥30 vs 18.5<BMI<25)	0.41(0.19–0.91)	0.96
Symptom duration (<5 years vs ≥5 years)	1.62(0.86–3.04)	0.13
Time since diagnosis (<5 years vs ≥5 years)	1.53(0.71–3.27)	0.28
CRP (≤ULN vs >ULN)	0.46(0.24–0.87)	0.02
MRI (negative vs positive)	0.75(0.37–1.54)	0.43
HLA-B27 (negative vs positive)	0.25(0.11–0.60)	0.002
MRI-/HLA-B27+ vs MRI+/HLA-B27+	0.58(0.27–1.26)	0.95
Swollen joint count >0 (no vs yes)	1.28(0.68–2.40)	0.44
MASES>0 (no vs yes)	2.73(1.18–6.32)	0.02
Prior DMARDs (no vs yes)	0.64(0.34–1.20)	0.16
Concomitant DMARDs at baseline (no vs yes)	0.46(0.23–0.91)	0.03
Prior use of NSAIDs (≤2 vs >2)	1.49(0.78–2.82)	0.22
Baseline ASDAS (greater vs smaller)	1.54(1.04–2.29)	0.03
Baseline BASDAI score (greater vs smaller)	1.06(0.85–1.33)	0.60
Baseline BASFI score (greater vs smaller)	0.88(0.76–1.03)	0.11
Baseline BASMI score (greater vs smaller)	0.67(0.51–0.87)	0.003
Baseline SI joint SPARCC score (greater vs smaller)	1.58(1.11–2.25)	0.01
Baseline PtGADA (greater vs smaller)	0.93(0.79–1.09)	0.36
Baseline total spinal pain score (greater vs smaller)	0.84(0.71–1.01)	0.06
Baseline nocturnal pain score (greater vs smaller)	0.90(0.78–1.03)	0.13
Baseline AsQoL (greater vs smaller)	0.58(0.15–2.15)	0.41

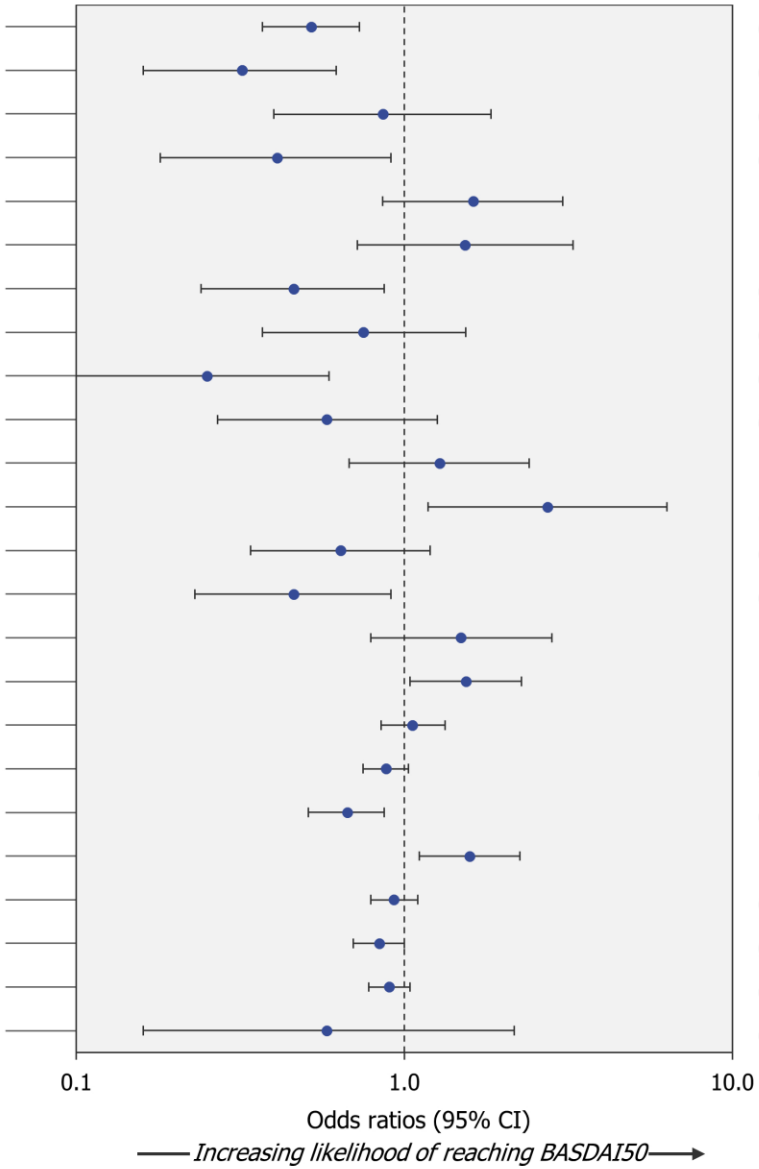

D) Predictive factors for ASDAS-ID

	Odds ratio (95% CI)	p-value
Age (older vs younger)	0.46(0.30–0.70)	<0.001
Gender (female vs male)	0.25(0.12–0.54)	<0.001
BMI (25≤BMI<30 vs 18.5<BMI<25)	0.98(0.44–2.17)	0.96
BMI (≥30 vs 18.5<BMI<25)	0.43(0.17–1.12)	0.97
Symptom duration (<5 years vs ≥5 years)	3.21(1.52–6.77)	0.002
Time since diagnosis (<5 years vs ≥5 years)	1.62(0.65–4.05)	0.30
CRP (≤ULN vs >ULN)	1.17(0.58–2.35)	0.66
MRI (negative vs positive)	0.47(0.19–1.16)	0.10
HLA-B27 (negative vs positive)	0.33(0.11–0.99)	0.05
MRI-/HLA-B27+ vs MRI+/HLA-B27+	0.39(0.16–0.98)	0.97
Swollen joint count >0 (no vs yes)	1.32(0.66–2.68)	0.43
MASES>0 (no vs yes)	2.61(1.18–5.78)	0.02
Prior DMARDs (no vs yes)	0.42(0.21–0.87)	0.02
Concomitant DMARDs at baseline (no vs yes)	0.42(0.21–0.87)	0.02
Prior use of NSAIDs (≤2 vs >2)	1.81(0.90–3.66)	0.10
Baseline ASDAS (greater vs smaller)	0.63(0.40–0.98)	0.04
Baseline BASDAI score (greater vs smaller)	0.66(0.50–0.87)	0.003
Baseline BASFI score (greater vs smaller)	0.67(0.55–0.81)	<0.001
Baseline BASMI score (greater vs smaller)	0.59(0.42–0.81)	0.001
Baseline SI joint SPARCC score (greater vs smaller)	1.42(1.05–1.92)	0.02
Baseline PtGADA (greater vs smaller)	0.72(0.59–0.87)	<0.001
Baseline total spinal pain score (greater vs smaller)	0.68(0.56–0.84)	<0.001
Baseline nocturnal pain score (greater vs smaller)	0.72(0.61–0.84)	<0.001
Baseline AsQoL (greater vs smaller)	0.07(0.02–0.33)	<0.001

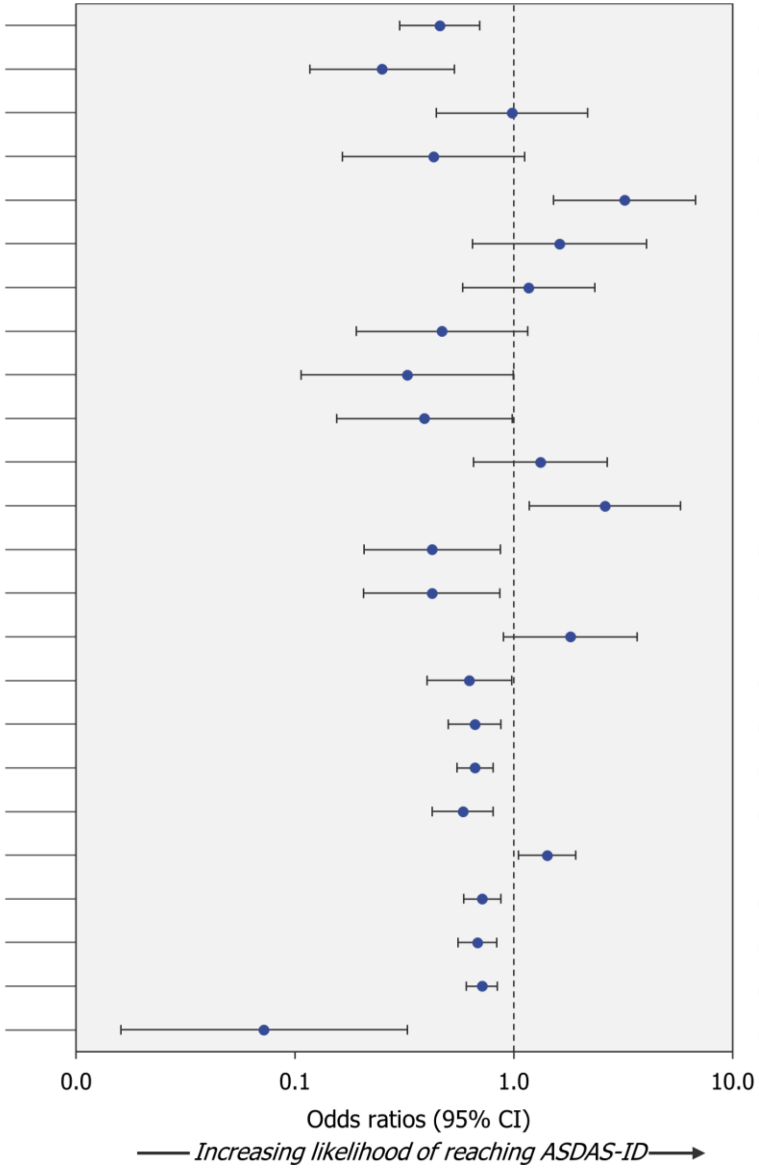

Full Analysis set (n=159). Patients received CZP 200 mg Q2W (400 mg loading dose at Weeks 0, 2 and 4) plus non-biologic background medication. Variables with 95% CI that were <0.0001 or >999.9 are excluded. 95% CI and p-values are considered non-confirmatory due to the exploratory nature of the analysis. ASAS40: Assessment of SpondyloArthritis International Society 40%; ASDAS: Ankylosing Spondylitis Disease Activity Score; ASDAS-ID: ASDAS inactive disease (ASDAS<1.3); ASDAS-MI: ASDAS major improvement (reduction in ASDAS≥2.0); ASQoL: ankylosing spondylitis quality of life; BASDAI50: Bath Ankylosing Spondylitis Disease Activity Index 50%; BASFI: Bath Ankylosing Spondylitis Functional Index; BASMI: Bath Ankylosing Spondylitis Metrology Index; BMI: body mass index; CI: confidence interval; CRP: C-reactive protein; CZP: certolizumab pegol; DMARD: disease-modifying antirheumatic drug; HLA-B27: human leukocyte antigen-B27; MRI+/-: presence/absence of sacroiliitis on magnetic resonance imaging; MASES: Maastricht Ankylosing Spondylitis Enthesitis Score (range 0−13); NRI: non-responder imputation; NSAID: non-steroidal anti-inflammatory drug; PtGADA: Patient Global Assessment of Disease Activity; Q2W: every 2 weeks; SPARCC: Spondyloarthritis Research Consortium of Canada; vs: versus.

Figure S3. Predictive factors of Week 52 response in CZP-treated patients who did not experience changes in background medication

A) Predictive factors for ASDAS-MI

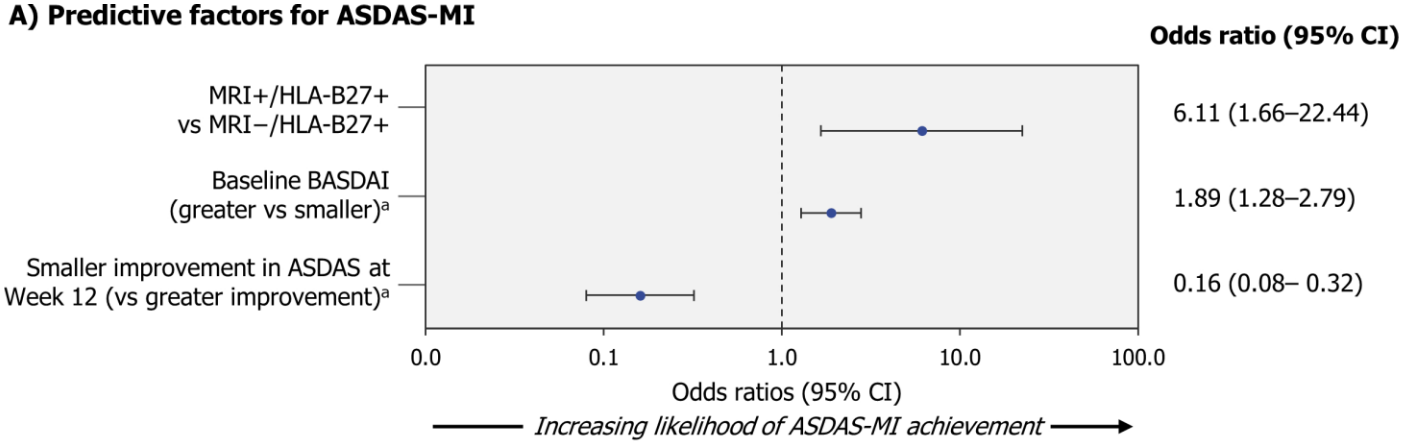

B) Predictive factors for ASAS40

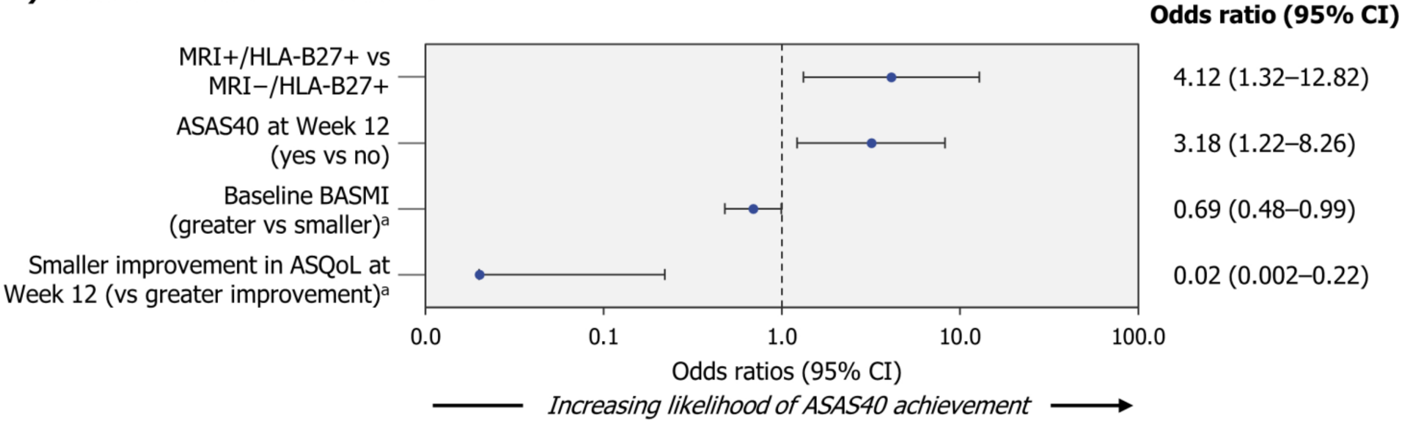

C) Predictive factors for BASDAI50

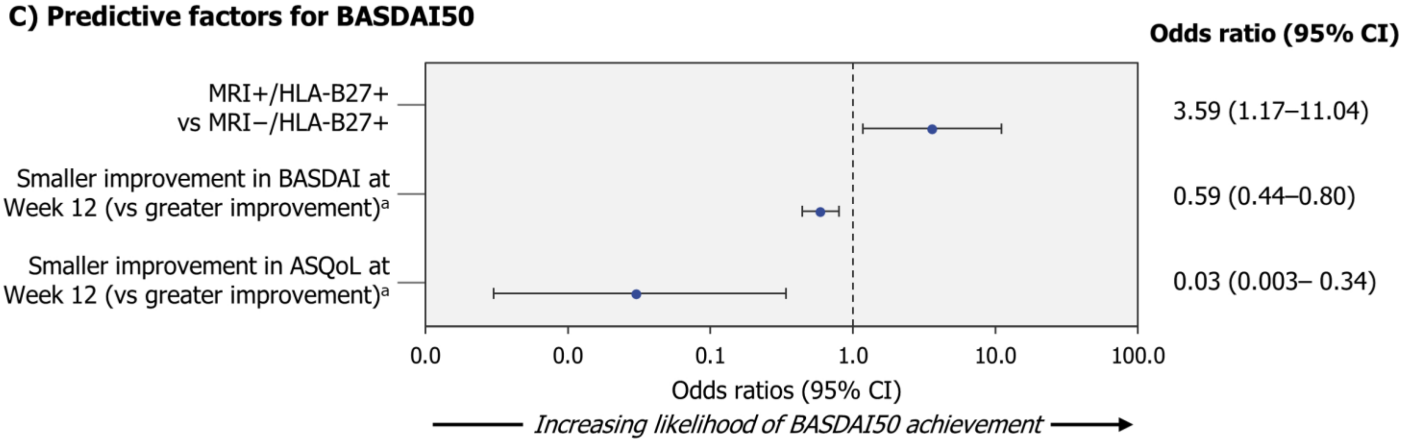

D) Predictive factors for ASDAS-ID

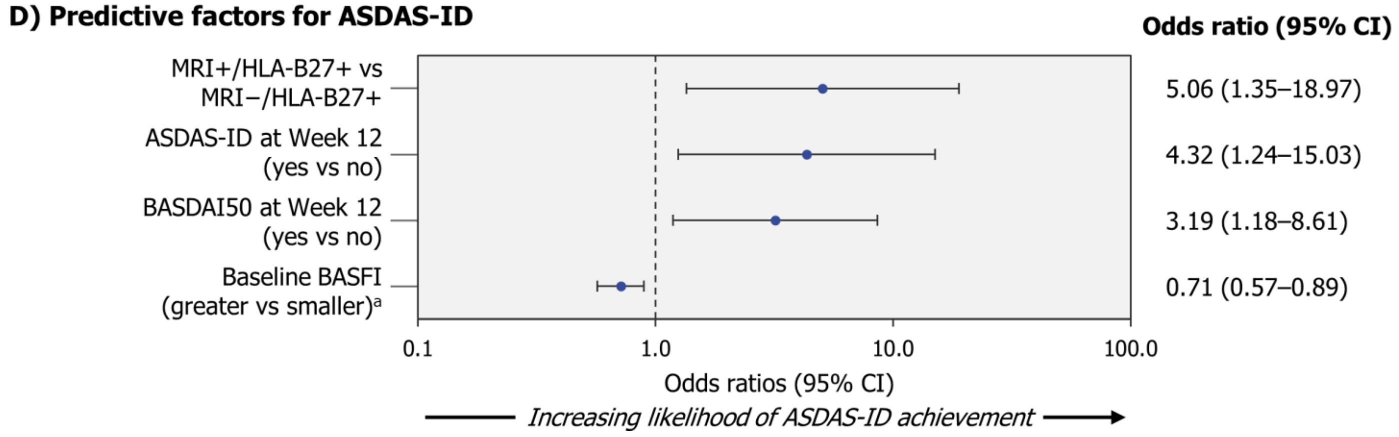

Randomised Set (NRI). Patients received CZP 200 mg Q2W (400 mg loading dose at Weeks 0, 2 and 4) plus non-biologic background medication. Analysis excludes patients who experienced any change in background medication. [a]Included in the predictive model as continuous variables; for these factors, an odds ratio >1 indicates a higher probability of larger values being predictive of a response. For Week 12 change from baseline measures, a lower (negative) value is indicative of improvement, while larger (positive) values indicate worsening. ASAS40: Assessment of SpondyloArthritis International Society 40%; ASDAS: Ankylosing Spondylitis Disease Activity Score; ASDAS-ID: ASDAS inactive disease (ASDAS<1.3); ASDAS-MI: ASDAS major improvement (reduction in ASDAS≥2.0); ASQoL: ankylosing spondylitis quality of life; BASDAI50: Bath Ankylosing Spondylitis Disease Activity Index 50%; BASFI: Bath Ankylosing Spondylitis Functional Index; BASMI: Bath Ankylosing Spondylitis Metrology Index; CI: confidence interval; CZP: certolizumab pegol; HLA-B27: human leukocyte antigen-B27; MRI+/-: presence/absence of sacroiliitis on magnetic resonance imaging; NRI: non-responder imputation; Q2W: every 2 weeks; vs: versus.
